# Quantum state processing through controllable synthetic temporal photonic lattices

**DOI:** 10.1038/s41566-024-01546-4

**Published:** 2024-10-14

**Authors:** Monika Dhinwa, Farzam Nosrati, Agnes George, Stefania Sciara, Riza Fazili, André Luiz Marques Muniz, Arstan Bisianov, Rosario Lo Franco, William J. Munro, Mario Chemnitz, Ulf Peschel, Roberto Morandotti

**Affiliations:** 1 Institute of Condensed Matter Theory and Optics, Friedrich-Schiller-University, Jena, Germany; 2 Institut national de la recherche scientifique, Centre Énergie Matériaux Télécommunications, Varennes, Quebec Canada; 3 Dipartimento di Ingegneria, Università degli Studi di Palermo, Palermo, Italy; 4 Fraunhofer Institute for Applied Optics and Precision Engineering, Jena, Germany; 5 Institute of Semiconductor Technology, Technical University of Braunschweig, Braunschweig, Germany; 6 Okinawa Institute of Science and Technology Graduate University, Okinawa, Japan; 7 Leibniz Institute of Photonic Technology, Jena, Germany; 8 Institute of Applied Optics and Biophysics, Abbe Center of Photonics, Friedrich-Schiller-University, Jena, Germany

**Keywords:** Quantum optics, Fibre optics and optical communications

## Abstract

Quantum walks on photonic platforms represent a physics-rich framework for quantum measurements, simulations and universal computing. Dynamic reconfigurability of photonic circuitry is key to controlling the walk and retrieving its full operation potential. Universal quantum processing schemes based on time-bin encoding in gated fibre loops have been proposed but not demonstrated yet, mainly due to gate inefficiencies. Here we present a scalable quantum processor based on the discrete-time quantum walk of time-bin-entangled photon pairs on synthetic temporal photonic lattices implemented on a coupled fibre-loop system. We utilize this scheme to path-optimize quantum state operations, including the generation of two- and four-level time-bin entanglement and the respective two-photon interference. The design of the programmable temporal photonic lattice enabled us to control the dynamic of the walk, leading to an increase in the coincidence counts and quantum interference measurements without recurring to post-selection. Our results show how temporal synthetic dimensions can pave the way towards efficient quantum information processing, including quantum phase estimation, Boson sampling and the realization of topological phases of matter for high-dimensional quantum systems in a cost-effective, scalable and robust fibre-based setup.

## Abstract (web summary)

A scalable quantum processor based on the discrete-time quantum walk of time-bin-entangled photon pairs on synthetic temporal photonic lattices is realized on a fibre-coupled loop system. Key fundamental quantum operations are demonstrated.

## Main

Entanglement and non-classical states are vital resources at the heart of many quantum technologies, including computation^1^, secure communication^2^, metrology^3^ and imaging^4^. Exploiting these resources requires phase-stable quantum circuitries^5,6^ delivering key operations, such as entanglement generation, quantum state manipulation and detection^7^. In this regard, quantum walks (QWs)^8–10^ have proven to be a promising framework for search^11^ and universal quantum computing^12^, among others^13^, which can be realized with a variety of quantum systems, including ion traps^14^, superconducting spins^15^ and photons^8,9^. In conventional all-optical QW platforms, the light flow (walk) is performed along optical paths that are connected in real-space interferometer meshes either in free space^16^ or in on-chip architectures^8,9,17–19^. Alternatively, QW implementations on large topologies can be offered by so-called synthetic photonic lattices^20^, which build on the intrinsic parallelism of optical operations at reduced device complexity. In a synthetic photonic lattice, the real space dimension—given by the optical path—is replaced by a photonic degree of freedom. Photonic modes that are mostly used to create synthetic dimensions are polarization^16^, orbital angular momentum^21^, frequency^22^ and time^23^.

Discrete temporal modes (also known as time bins^24–27^) offer an ideal platform to create synthetic scalable dimensions, due to the several advantages they bring about, including noise robustness, as well as room-temperature manipulation of multiple time modes in single fibre channels and compatibility with standard optical telecommunications architecture. In temporal photonic lattices (TPLs)^28^, time bins are typically realized via temporal delays between optical pulses^29^ and can be implemented using off-the-shelf fibre technologies. TPLs offer excellent testbeds to simulate intriguing effects, such as parity–time symmetry^28,30^, superfluidity of light^31^ and topological structures^32^. However, despite multiple proposals to use TPLs for quantum information processing^33,34^, single- and few-photon-based experimental implementations have been hampered by limitations in current photonic devices, such as gate inefficiencies.

Here, we show how TPLs can be employed for practical and scalable quantum information processing based on a discrete-time quantum walk (DTQW)^12,35,36^ of time-bin-entangled photon states. We realize the TPL on a fully fibre-based coupled-loop system, which is used for two-photon state preparation, manipulation and quantum interference measurements. Building on the dynamic control over the quantum walk and circuitry, we can achieve two-level quantum interference without recurring to post-selection, as well as four-level quantum interference at enhanced detection efficiencies.

**Fig. 1:**
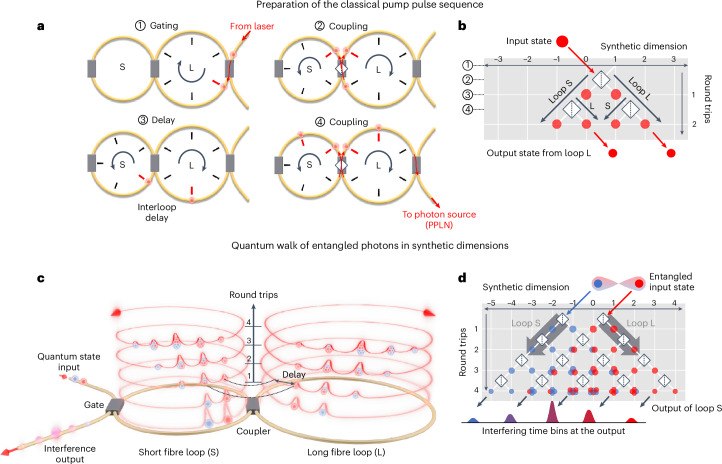
Illustration of entangled state preparation and quantum interference with the coupled fibre-loop system. **a**, A schematic of the four-step process to generate a double-pulse sequence from a single laser pulse. Optical modes can be dynamically gated in and out of each loop. Labels S and L represent the short and long fibre loops, respectively, with ultrafast central couplers in between to couple them. Angular grids inside the loops show the position of the time bins with ~100 ns delay. **b**, The corresponding spatial network with synthetic position *n* in the lattice and the number of roundtrips *m*. It illustrates two roundtrips resulting in four pulses (two per loop) from a single pulse. A step to the left (right) corresponds to light propagation in short (long) loops. **c**, An artistic depiction of light propagation in the fibre-loop system for a few roundtrips. Light spreads along the synthetic temporal space created by the interloop delay with an increasing number of roundtrips (depicted as different five-level spirals). **d**, The corresponding DTQW network. With each roundtrip, the complexity of the modal contribution to each time bin, indicated by the colour-coded bullets in both **c** and **d**, increases, resulting in different quantum interferences.

**Fig. 2:**
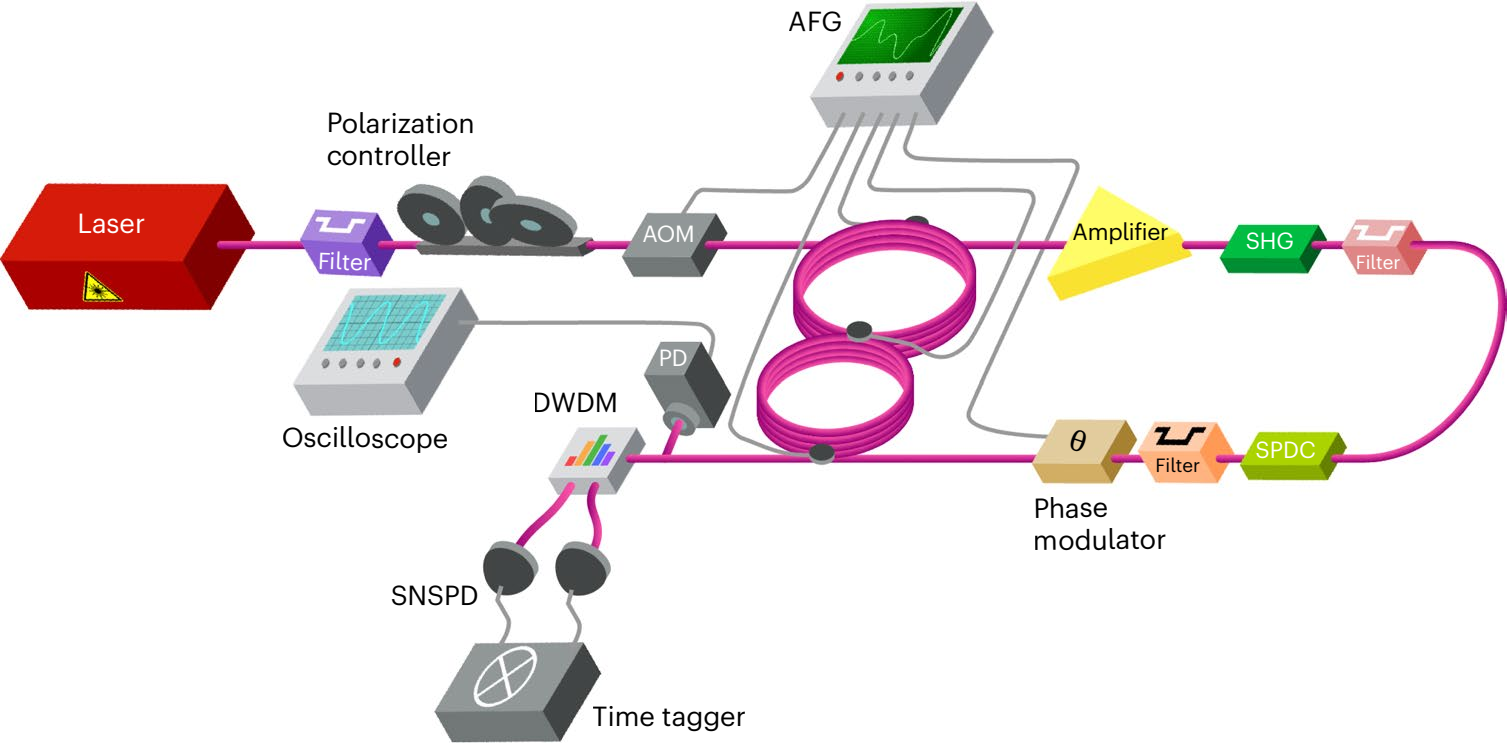
Experimental setup. A coupler connects two polarization-maintaining fibre loops of 120 m (600 ns) for the long loop and 100 m (500 ns) for the short loop. AFGs dynamically control the coupler’s transmittance. The setup also includes a pulsed laser; an acousto-optic modulator (AOM) to reduce the repetition rate of the laser; PPLN nonlinear crystals to generate photons through SHG and SPDC; phase modulator; wavelength demultiplexer (WDM); oscilloscope; photodetector (PD); and two SNSPDs. The oscilloscope and PDs are used in the classical part of the experiment.

In our experiment, we operate on a one-dimensional synthetic TPL created through an unbalanced fibre-loop system with a difference of δ*l* = 20 m between the length of the two loops. Light can enter the system at defined modes (that is, lattice grid points) in one loop (the long one) through an optical switch (gate) and then dynamically couple to the other loop through an ultrafast optical variable central coupler that acts as a dynamic interconnect between the loops^23^ (Methods). As pulses propagate through the two loops, they arrive at the coupler at different times (Fig. 1a) delayed by *τ* = 100 ns, and hop to their next-neighbouring mode at the upcoming roundtrip. In other words, the delay between the pulses propagating within the two loops is equivalent to a transverse dimension in a conventional photonic mesh lattice resembling a spatial QW model^10^, as illustrated in Fig. 1b for two roundtrips. Depending on the targeted operation, the pulses remain in the loops for two (or four) roundtrips to generate two (or four) classical pulses. The steps to prepare a classical double pulse sequence in our practical loop-based implementation and its corresponding representation in the synthetic space are depicted in Fig. 1a,b, respectively. These pulses are then taken out from the long loop (via the optical switch shown in Fig. 1a) and pass through a series of two periodically poled lithium niobate (PPLN) nonlinear waveguides^37,38^ to generate two-level (or four-level) time-bin-entangled photon pairs via spontaneous parametric downconversion (SPDC; Fig. 2 and Methods). As illustrated in Fig. 1c,d, the time bins are considered as the initial quantum state and sent back to the short loop (via the optical switch) to perform quantum interference measurements.

The state of a quantum walker can be defined as $|{\psi }\rangle =\sum {\alpha }_{\kappa }|\kappa \rangle$, where *α*_*κ*_ is the probability amplitude of finding the walker at the mode $|{{\kappa }}\rangle$, while $|{{\kappa }}\rangle \,:=\,{\left|n\right\rangle }_{{{{\mathrm{p}}}}}{\left|\sigma \right\rangle }_{{{{\mathrm{c}}}}}$, with p and c labelling the position and coin states of the walker, respectively. In our platform, the coin state ${\left|\sigma \right\rangle }_{{{{\mathrm{c}}}}}=\,\{|{\rm{S}}{\rangle }_{{{{\mathrm{c}}}}},|{\rm{L}}{\rangle }_{{{{\mathrm{c}}}}}\}$ is given by the short ($|{\rm{S}}\rangle$) and the long ($|{\rm{L}}\rangle$) paths in the loop, while the synthetic position ${\left|n\right\rangle }_{{{{\mathrm{p}}}}}$ is given by the time bin. The whole evolution of a quantum walker can be attributed to the repeated action of a unitary operator resulting from the product of the position shift and the coin operators (Methods and Supplementary Section 1). A unique feature of our all-fibre design is the dynamic tuneability of the reflectance and transmittance ratio enabled by the ultrafast dynamic (central) coupler positioned between the two fibre loops. The coupler further enables transitions from full transmission ($\widehat{T}$) to both 50:50 splitting ($\widehat{F}$) and full reflection ($\widehat{R}$) within 50 ns, that is, half of the spacing between the time bins.

We used this ‘dynamic’ TPL to prepare and process time-bin-entangled photon pairs via DTQW. In the preparation stage, we inject a single laser pulse into the TPL through the long loop. After *d* roundtrips, we obtain a sequence of *d* pulses, used to pump a cascade of two PPLN waveguides (Fig. 2). As a result, we generate *d*-level photon pairs of the form^39^ $|{{\psi }}_{{\mathrm{d}}}\rangle =\frac{1}{\sqrt{d}}{\sum }_{t=0}^{d-1}{{\mathrm{e}}}^{2{{{{i}}}}t\theta }{|t\rangle }_{{\mathrm{s}}}{|t\rangle }_{{\mathrm{i}}}$ with *d* = 2 or 4, *s* and *i* respectively denoting the signal and idler photons, *θ* being the relative phase between consecutive time bins and *t* indicating the time bin. In this notation, ‘level’ refers to the number of temporal modes encoded per photon. Here, the time width is 7 ps (corresponding to an ~150 GHz bandwidth), while the temporal delay between the bins is *τ* = 100 ns. The generated entangled states are transferred back into the TPL through the optical switch in the short loop to tune their evolution. The photons are finally taken out from the fibre-loop system via the short loop and routed each to two different channels of a superconducting nanowire single-photon detector (SNSPD) system for coincidence counting (Fig. 2).

We utilize the TPL to implement projections measurements of the form^39^

1 $$|{{\psi }}_{{\rm{p}}}\rangle =\frac{1}{d}\left(\mathop{\sum }\limits_{t=0}^{d-1}{{\mathrm{e}}}^{{{i}}t\theta }{|t\rangle }_{{\mathrm{s}}}\right)\left(\mathop{\sum }\limits_{t=0}^{d-1}{{\mathrm{e}}}^{{{i}}t\theta }{|t\rangle }_{{\mathrm{i}}}\right)$$

to realize quantum interference (Methods), with the subscript p labelling the projection. These are accomplished by using both ‘uncontrolled’ and ‘controlled’ DTQW schemes. In the uncontrolled configuration, the central coupler is maintained at a fixed 50:50 ratio throughout the experiment, while, in the controlled case, it is dynamically switched at different coupling ratios (full reflection, full transmission and 50:50). In other words, the central coupler is tuned during each roundtrip (*m*) and at each synthetic position (*n*) (Methods).

**Fig. 3:**
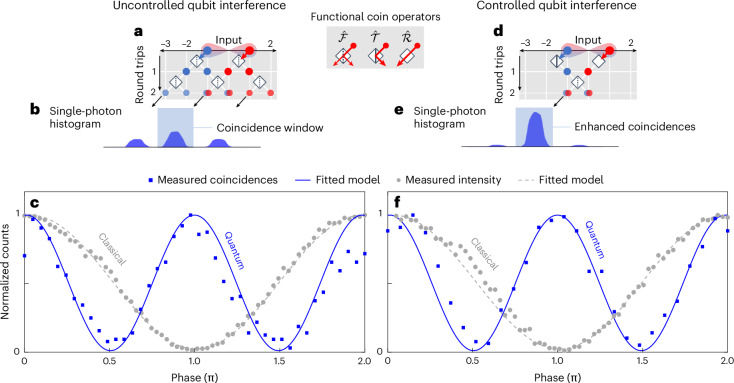
Two-level quantum interferences. **a**,**d**, Spatial representation of two roundtrips of the DTQW in the coupled fibre-loop system under uncontrolled (**a**) and controlled (**d**) schemes. The legend in the centre introduces the action of the three operators used in the experiment (Fourier (50:50), transmission and reflection). **b**,**e**, Single-photon histograms obtained through the uncontrolled (**b**) and controlled (**e**) schemes. The light-blue box in the single-photon histogram represents the interference coincidence window at zero relative time delay taken from the central interference bin. **c**,**f**, Normalized coincidence counts of entangled photons and classical light intensity (blue squares and grey circles, respectively) as a function of the relative phase difference (*θ*) between the time bins, yielding two-photon quantum interference patterns for the uncontrolled (**c**) and controlled (**f**) schemes. Experimental measurements are fitted with theoretical models (blue solid line for entangled qubits and dashed grey line for classical light). From two-photon quantum interference measurements, raw visibility values of *V*_*d*=2_ = 97.82% and *V*′_*d*=2_ = 96.83% were extracted for the uncontrolled (**c**) and controlled (**f**) schemes, respectively.

We first utilize the uncontrolled scheme to perform quantum interference measurements of two-level (qubits) time-bin-entangled photon pairs. To this end, we inject/transfer the two-photon state $\left|{\psi }_{2}\right\rangle =\frac{1}{\sqrt{2}}({\left|-1{\rm{S}}\right\rangle }_{{\mathrm{s}}}{\left|-1{\rm{S}}\right\rangle }_{i}+{\mathrm{e}}^{2{{i}}\theta }{\left|1{\rm{S}}\right\rangle }_{{\mathrm{s}}}{\left|1{\rm{S}}\right\rangle }_{{\mathrm{i}}})$, where, for example, the notation ${\left|-1{\rm{S}}\right\rangle }_{{\mathrm{s}}}$ represents the state of the signal photon in the synthetic position *n* = −1 and in the coin state ${\mathrm{S}}$ (that is, the short loop) of the TPL, and so forth for the other terms. We thus let the photons propagate for two roundtrips, as schematically shown in Fig. 3a. Upon post-selecting the coincidence events between the photons extracted from the short loop, as shown in Fig. 3b, we measure quantum interference as a function of the phase *θ*, yielding a raw visibility of *V*_*d*=2_ = 97.82%, which exceeds the threshold of 70.71% required to violate the Bell inequality^40^ (Fig. 3c). We then repeat quantum interference measurements by applying the controlled DTQW scheme on the QW. As schematically described in Fig. 3d, the quantum state evolution is realized by directing the photon(s) in the earlier time bin (position *n* = −1) for the long loop and by fully reflecting the photon(s) in the later time bin (position *n* = +1) for the short loop (S) using the dynamic central coupler. The delay experienced by the time bins in the second roundtrip allows them to coherently interfere at the central coupler, where they both arrive simultaneously. Setting the coupler at a 50:50 ratio results into only one bin in each loop. We then measure coincidences between the photons extracted from the short loop as a function of the phase *θ* across the entire gated time window (550 ns) of the SNSPDs, meaning that no post-selection is applied to remove the side peaks (Fig. 3e). Fitting the expected interference response to the data yields a raw visibility (that is, including background noise) of *V*′_*d*=2_ = 96.83%, as shown in Fig. 3f. We also repeat the same measurement with classical light and compare the obtained normalized light intensity with the quantum interference pattern. The controlled DTQW scheme enables quantum interference without any noise subtraction with maximum detection efficiency ${p}_{0}=1$ (Methods), which can be explained as follows. In this scheme, the presence of side bins is, ideally, avoidable. In this experiment, only negligible peaks appear on the sides, as shown in the single-photon histogram in Fig. 3e. The residual counts can be attributed to photons’ leakage due to the non-ideal behaviour of both the central coupler and the gate. It is noteworthy that these photons do not contribute to the coincidence counts, which means that post-selection is avoidable in this case. In contrast, the uncontrolled DTQW scheme exhibits a significantly lower detection probability of ${p}_{0}\,=\,1/4$ (Supplementary Section 3) due to the distribution of the photons into three time bins (Fig. 3b). As a result, the controlled DTQW scheme enables an increase of the coincidence counts from 21 to 77 per minute with respect to the uncontrolled counterpart.

A peculiarity of the TPL demonstrated therein is its scalability to higher-level time bins. Increasing the dimensionality of the temporal modes typically requires multiple-arm unbalanced interferometers to perform multi-level projections for quantum state processing. However, stabilizing multiple-arm fibre interferometers can be highly resource-demanding, time-consuming and overall impractical. Our system bypasses the need for multiple-arm interferometers. Here, we demonstrate the scalability of TPLs by generating and processing four-level time-bin-entangled photon pairs. The laser pulse was sent into the fibre-loop system (via the optical switch in the long loop) to prepare a sequence of four pulses, so as to pump the PPLN waveguides. The generated entangled photon pairs were then injected back into the TPL through the short loop in the form $\left|{\psi }_{4}\right\rangle =\frac{1}{2}\left({\left|-3{\rm{S}}\right\rangle }_{{\mathrm{s}}}{\left|-3{\rm{S}}\right\rangle }_{{\mathrm{i}}}+{\mathrm{e}}^{2{{i}}\theta }{\left|-1{\rm{S}}\right\rangle }_{{\mathrm{s}}}{\left|-1{\rm{S}}\right\rangle }_{{\mathrm{i}}}\right.$ $\left.+{{\mathrm{e}}}^{4{{i}}\theta }{\left|1{\rm{S}}\right\rangle }_{{\mathrm{s}}}{\left|1{\rm{S}}\right\rangle }_{{\mathrm{i}}}+{{\mathrm{e}}}^{6{{i}}\theta }{\left|3{\rm{S}}\right\rangle }_{{\mathrm{s}}}{\left|3{\rm{S}}\right\rangle }_{{\mathrm{i}}}\right)$, where we adopted the same notation as for the state $|{\psi }_{2}\rangle$.

**Fig. 4:**
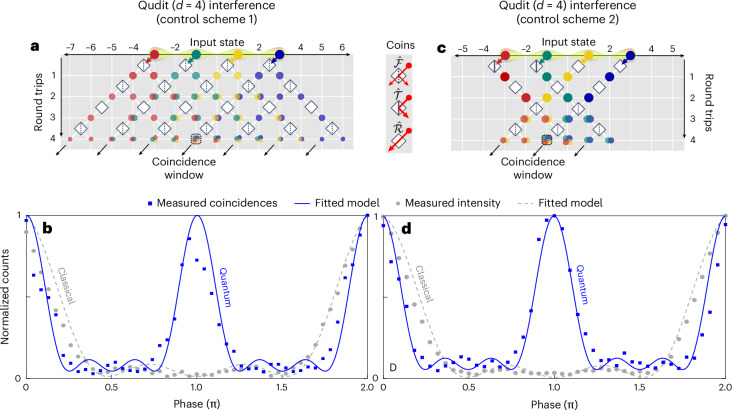
Four-level quantum interferences. **a**,**c**, Spatial representation of four roundtrips of the DTQW in the coupled fibre-loop system under a roundtrip-wise controlled scheme (scheme 1) (**a**) and roundtrip- and synthetic position-wise controlled scheme (scheme 2) (**c**). Coincidence counts are measured at the central time bin coincidence window of the fibre loop’s interference output, and at zero photon time delay. **b**,**d**, Normalized coincidence counts of four-level entangled photons and classical light intensity (blue squares and grey circles, respectively) as a function of the relative phase difference (*θ*) between the time bins. Experimental measurements are fitted with theoretical models (solid blue line for entangled qudits and dashed grey line for classical light). From two-photon quantum interference measurements, raw visibility values of *V*_*d*=4_ = 91.55% and *V*′_*d*=4_ = 89.61% were extracted for scheme 1 (**b**) and scheme 2 (**d**), respectively.

To implement two-photon four-level quantum interference measurements, we employed two controlled DTQW strategies, namely, inter-roundtrip and intra-roundtrip control schemes (referred herein as scheme 1 and scheme 2, respectively). In control scheme 1, depicted in Fig. 4a, the central coupler is fixed in a specific configuration within the same roundtrip, while it is dynamically tuned into a different configuration between roundtrips. In control scheme 2, depicted in Fig. 4c, the central coupler is dynamically tuned in different configurations both within the same roundtrip (that is, between synthetic positions) and between roundtrips (see Supplementary Sections 1 and 3 for more details on step-by-step operation). Figure 4b,d shows two-photon quantum interference measurements obtained through both control schemes. Coincidences were measured for different phases *θ* by extracting the two photons from the short loop. Control scheme 2 allows for an enhancement in the coincidence counts, specifically from 4.5 counts per minute for scheme 1 to 8.3 counts per minute for scheme 2 (see Supplementary Section 3 for more theoretical details). We extracted raw visibility values of *V*_*d*=4_ = 91.55% and *V*′_*d*=4_ = 89.61% for control scheme 1 and 2, respectively. These values exceed the visibility threshold necessary to violate the Collins–Gisin–Linden–Massar–Popescu inequality^41–43^ (81.70%; Supplementary Section 2). The higher degree of control in scheme 2 comes at the price of higher losses introduced in the system. This justifies the lower visibility value measured through that scheme as compared with scheme 1. We also repeat the same measurement with classical light and compare the obtained normalized light intensity with the two-photon quantum interference pattern (see grey data points in Fig. 4b,c).

We demonstrate the quantum properties of temporal photonics lattices as a promising framework for the preparation, generation and manipulation of *d*-level time-bin-entangled photon pairs based on a dynamically coupled fibre-loop system and DTQW. We could implement a controlled QW scheme enabling us to measure quantum interference for two-level entangled states without recurring to post-selection, as well as to optimize the evolution of the quantum walker for higher detection efficiencies and coincidence counts for both two- and four-level cases. Quantum interference measurements without post-selection can only occur if a single time bin, containing all necessary information, is present throughout the detection window. In the two-level case, it was possible to reduce all the three interfering bins into one, while, in the four-level case, it was not possible to decrease the number of time bins to less than 3 owing to the given number of roundtrips, which made it necessary to post-select the central interference peak.

The proposed measurement strategy, based on TPLs, can be theoretically generalized to perform quantum interference, as well as to maximize detection efficiency for any number of levels (time bins; Methods and Supplementary Section 4). Hence, the proposed approach may inspire new designs for engineering TPLs capable of generating, processing and detecting entangled modes with arbitrarily high dimensions at maximal efficiencies.

In particular, we show how temporal synthetic photonic lattices can be used on a fully fibre-based coupled-loop system for quantum experiments, specifically for the preparation and processing of high-dimensional time-bin-entangled photon pairs. This is achieved by mapping the temporal synthetic photonic lattice to the DTQW formalism, which allows for the control of the evolution of the QW network, leading to optimized quantum interference measurements. Moreover, our entire implementation is fibre-based and operative in the telecom range. As such, we expect our system to be combined with current and future telecom infrastructures towards enhanced quantum information processing for quantum networks, such as phase estimation problems (Supplementary Section 5). The controllability of our design boosts coincidence counts, which is vital for applications like quantum key distribution protocols where measurement control and count rate increase are key to enhancing secret key rates^44^. Indeed, a faster optical coupler (~1 ns response time) with lower injection losses («1 dB) will be pivotal to boost the technological potential of our scheme. It can indeed lead to an increase in the control speed and, hence, the number of modes and achievable roundtrips. Given these improvements, our work suggests that TPLs based on synthetic scalable dimensions have the potential to support a variety of quantum information protocols, such as entanglement transfer^45^, Boson sampling^33,46^, state estimation/discrimination^47,48^ and quantum metrology^49^.

## Methods

### Experimental setup

The sketch of the experimental setup used to realize the synthetic dimension is shown in Fig. 2. This setup is used for generating the time-bin-entangled photon pairs from classical light pulses and for performing quantum state projections. Initially, a single pulse from a femtosecond fibre laser source (PriTel Femtosecond Fiber Laser series, 1,550 nm wavelength, 10 MHz repetition rate and timing jitter <1 ps) is sent into the fibre-loop system after its repetition rate and bandwidth are reduced to 181.8 kHz and 0.3 nm using an acousto-optic modulator (AOM, 40 dB suppression ratio) and a tunable filter, respectively. Due to the length of the fibre loops (~100 m (500 ns) and 120 m (600 ns)), reducing the repetition rate is necessary to have sufficient time to complete the measurement procedure. Pulses and time bins are injected and taken out from the fibre-loop system using optical switches (gates) (Nanona optical switcher from BATi). The short and the long loops are connected via a dynamic (central) coupler (Nanona optical switcher from BATi). Both switches and coupler are precisely controlled via arbitrary function generators (AFGs by Agilent 33600A). All control patterns could be realized via rectangular waveforms with outputs of 0 V (gate closed or coupler in full reflection), 1.16 V (50:50 splitting ratio) and 2.6 V (gate open or coupler in full transmission). The AFG is also used to drive a phase modulator after the photon source (Fig. 2), which is utilized to apply and tune the relative phase *θ* between the temporal entangled modes, that is, two successive time bins, as necessary for quantum interference measurements. This results in a phase shift of 2*θ* for the two-photon state. The light pulse injected into the fibre-loop system through the optical switch located in the long loop is then split by the central coupler into two pulses, one circulating in the longer and the other in the shorter loop. The pulses return to the central coupler with a 100 ns temporal delay between them before they are split again by the coupler. Repeating this process in each roundtrip eventually leads to the generation of a *d*-fold burst in each loop.

In the next step, pulses are taken out from the long loop to pump a cascade of two PPLN nonlinear waveguides (embedded in the fully fibre-integrated system and commercially available; here, we use SRICO, model 2000-1547). An Erbium-doped fibre amplifier (Keopsys model PEFA-SP-C-PM-27-B202-FA-FA) was used to increase the peak power of the prepared classical pulse sequence from the fibre loop to enhance the power injected into the PPLN waveguides for subsequent photon state generation. An isolator ensured the rejection of backpropagating noise photons from this amplifier to the loop system. The first PPLN up-converts the classical pump pulses at 1,550 nm to an intense pulse sequence at 775 nm through the second harmonic generation (SHG) process within a 0.5 nm conversion bandwidth. Residual pump photons at 1,550 nm are filtered out after the up-conversion by a 90 dB-rejection short-pass edge filter (by Lightwave). The generated signal pulse train at 775 nm is then used to induce a probabilistic photon pair generation in a second PPLN waveguide. Here, SPDC converts a photon at 775 nm into two twin photons within 1,530 nm and 1,570 nm bandwidths. Due to the temporally modulated pumping and energy-momentum conservation of the SPDC process, both photons are mutually entangled in discrete time bins defined by the pump pulse sequence (two and four pulses for the two-level and four-level cases, respectively). Subsequently, residual pump light at 775 nm is filtered by the 90 dB-rejection long-pass edge filter and the entangled photon pairs are launched into the fibre loop for quantum state processing. Finally, photon pairs are generated in a time-bin-entangled state of the form $\left|{\psi }_{{\mathrm{d}}}\right\rangle =\frac{1}{\sqrt{d}}\mathop{\sum }\nolimits_{t=0}^{d-1}{{\mathrm{e}}}^{2{{i}}t\theta }{\left|t\right\rangle }_{{\mathrm{s}}}{\left|t\right\rangle }_{{\mathrm{i}}}$, where, for the cases analysed here, *d* = 2 or 4. These dimensionalities are given by the injection of two- and four-folded bursts after two and four roundtrips, respectively. This results into a two-photon entangled state, where each photon is in a superposition of two and four time modes, respectively. The generated and phase modulated entangled photon pairs are reinserted into the loop through the optical switch located in the short loop for quantum interference measurements.

After they have interfered, signal and idler photons are separated by a dense wavelength division multiplexer (DWDM, by Lightwave) at ∼1,553 nm and 1,547 nm, respectively, and then routed onto two SNSPDs (Opus One by Quantum Opus). A time tagger (by PicoQuant HydraHarp 400) records timing events and is used for coincidence measurements.

### Uncontrolled DTQW scheme

In the uncontrolled configuration, the coupler was maintained at a fixed 50:50 splitting ratio throughout the experiment. We used classical light for setup alignment and sent two (or four) pulses directly to the loop system without passing through the PPLNs. After the required number of roundtrips, we obtained the data and transmitted them to classical detectors, repeating this procedure for a range of phase values. Subsequently, we performed quantum measurements by sending a prepared quantum state into the loop system. From the single-photon histograms obtained from the SNSPDs, we identified different bins and measured coincidence counts within a specified window, repeating this procedure for a range of phase values. In the case of the two bins, the measurement of each phase point took ~3 min. Subsequently, we utilized equation (2) to perform curve fitting and determine the visibility, as demonstrated in Fig. 3c of the main text.

### Controlled DTQW scheme

In the controlled operations, the measurement procedure resembled that of the uncontrolled case, with the difference that the central coupler was dynamically controlled instead of being fixed at 50:50. To this end, we generated (via MATLAB) precise waveforms for different schemes, which were then transferred to the central coupler as per the desired application. Also here, we carried out measurements using classical light for alignment purposes, before conducting quantum experiments.

In the case of two time bins, the controlled configuration led to a significant decrease in photon counts outside the coincidence window, compared with the uncontrolled scheme, as evidenced by the single-photon histograms shown in Fig. 3b,e. While no temporal filtering was required to measure the coincidence counts, the number of coincidences remained the same for a specific coincidence window and the entire roundtrip window. Each phase point measurement had a duration of approximately 1 min. Finally, we utilized equation (2) to fit the curve and determine the visibility, with the results shown in Fig. 3f of the main text. In the case of four time bins, it was necessary to conduct deterministic measurements to perform quantum interference. Two different schemes were investigated: a roundtrip-wise controlled scheme (scheme 1) and a roundtrip- and synthetic position-wise controlled scheme (scheme 2). The controlled scheme 1 resulted into a low count in the coincidence window (still sufficient to draw conclusions), while the controlled scheme 2 led to a lower number of bins (here, three) after the same four roundtrips and a higher count in the coincidence window. Each phase point measurement took 60 min, and curve fitting using equation (3) was performed to determine visibility. The results are shown in Fig. 4b,d, respectively.

### Overall performance efficiency

The loss per gate mainly determines the maximum number of propagation steps in the walk. Considering that the photons pass through the coupler (~0.7 dB), the fibre (splice losses ~0.44 dB, transmission losses negligible) and one gate (~0.7 dB) in each roundtrip (that is, a propagation step), the overall losses per roundtrip total to 1.84 dB. An additional 0.7 dB needs to be added for the out-coupling. In the present experiments, a minimum of two roundtrips is needed for qubit processing and four roundtrips for the four-level qudit processing, amounting to 4.38 dB and 8.06 dB in total losses, respectively.

### Theoretical considerations of quantum interference measurement

We performed biphoton quantum interference measurements to extract the visibility associated with *d*-level states of the form $\left|{\psi }_{{{\mathrm{d}}}}\right\rangle =\frac{1}{\sqrt{d}}\mathop{\sum }\nolimits_{t=0}^{d-1}{{\mathrm{e}}}^{2{{i}}t\theta }{\left|t\right\rangle }_{{\mathrm{s}}}{\left|t\right\rangle }_{{\mathrm{i}}}$ with *d* = 2 or 4. The intended quantum interference measurement is given by the operator ${\hat{E}}_{\mathrm{p}}=\left|{\psi }_{\mathrm{p}}\right\rangle \left\langle {\psi }_{\mathrm{p}}\right|$ (see equation (1)), which ideally results in ${{\mathcal{P}}_{\mathrm{d}}}=\left|\left\langle {\psi }_{\mathrm{d}}|{\psi }_{\mathrm{p}}\right\rangle \right|^{2}$. We implemented the measurements by making the two photons propagate according to a controlled unitary evolution for $m$ roundtrips, which is ${|\psi }_{\mathrm{d}}\left(m\right)\rangle ={\varPi }_{j=1}^{m}{\hat{{\mathcal{U}}}}_{j}{\psi }_{\mathrm{d}}\left(0\right)$, where ${\hat{{\mathcal{U}}}}_{j}$ is the controllable unitary evolution at roundtrip $j$. Ultimately, we choose a proper coincidence window between the signal and the idler photons, leading to the expected biphoton quantum inference patterns given by^39^

2 $${{\mathcal{P}}_{2}}\left({\epsilon }_{2}\right)=\frac{1}{4}\left(1+{\epsilon }_{2}\cos 2\theta \right),$$

3 $${\mathcal{P}}_{4}\left({\epsilon }_{4}\right)=\frac{1}{{2}^{6}}\left(4+2{\epsilon }_{4}\left(3\cos 2\theta +2\cos 4\theta +\cos 6\theta \right)\right).$$

Here, assuming the white noise model, ${\epsilon }_{2}$ and ${\epsilon }_{4}$ are the probability for the quantum state to be affected by noise and must take values larger than ${\epsilon }_{2}=0.7071$ for *d* = 2 and ${\epsilon }_{4}=0.8170$ for *d* = 4 to violate the Bell and the Collins–Gisin–Linden–Massar–Popescu inequalities for two and four levels, respectively.

## Online content

Any methods, additional references, Nature Portfolio reporting summaries, source data, extended data, supplementary information, acknowledgements, peer review information; details of author contributions and competing interests; and statements of data and code availability are available at 10.1038/s41566-024-01546-4.

## Supplementary information

Supplementary Information Supplementary Section 1. DTQW. Supplementary Section 2. QW as a measurement device. Supplementary Section 3. Uncontrolled and controlled DTQW evolutions to perform two- and four-level biphoton quantum interference measurements. Supplementary Section 4. Two-photon quantum interference for arbitrary discrete dimensions using TPLs. Supplementary Section 5. An exemplary application of TPLs: single phase estimation via synthetic TPLs.

## Data Availability

The datasets supporting the findings of this work are available from the corresponding authors upon reasonable request.

## References

[1] Knill, E., Laflamme, R. & Milburn, G. J. A scheme for efficient quantum computation with linear optics. *Nature* **409**, 46–52 (2001). 11343107 10.1038/35051009

[2] Nadlinger, D. P. et al. Experimental quantum key distribution certified by Bell’s theorem. *Nature* **607**, 682–686 (2022). 35896644 10.1038/s41586-022-04941-5

[3] Giovannetti, V., Lloyd, S. & MacCone, L. Advances in quantum metrology. *Nat. Photonics* **5**, 222–229 (2011).

[4] Gilaberte Basset, M. et al. Perspectives for applications of quantum imaging. *Laser Photonics Rev.* **13**, 1900097 (2019).

[5] Wang, J., Sciarrino, F., Laing, A. & Thompson, M. G. Integrated photonic quantum technologies. *Nat. Photonics* **14**, 273–284 (2020).

[6] Arrazola, J. M. et al. Quantum circuits with many photons on a programmable nanophotonic chip. *Nature* **591**, 54–60 (2021). 33658692 10.1038/s41586-021-03202-1 PMC11008968

[7] Chen, X., Fu, Z., Gong, Q. & Wang, J. Quantum entanglement on photonic chips: a review. *Adv. Photonics* **3**, 064002 (2021).

[8] Peruzzo, A. et al. Quantum walks of correlated photons. *Science* **329**, 1500–1503 (2010). 20847264 10.1126/science.1193515

[9] Perets, H. B. et al. Realization of quantum walks with negligible decoherence in waveguide lattices. *Phys. Rev. Lett.* **100**, 170506 (2008). 18518267 10.1103/PhysRevLett.100.170506

[10] Venegas-Andraca, S. E. Quantum walks: a comprehensive review. *Quantum Inf. Process.* **11**, 1015–1106 (2012).

[11] Childs, A. M. & Goldstone, J. Spatial search by quantum walk. *Phys. Rev. A* **70**, 022314 (2004).

[12] Lovett, N. B., Cooper, S., Everitt, M., Trevers, M. & Kendon, V. Universal quantum computation using the discrete-time quantum walk. *Phys. Rev. A* **81**, 042330 (2010).

[13] Kadian, K., Garhwal, S. & Kumar, A. Quantum walk and its application domains: a systematic review. *Comput. Sci. Rev.* **41**, 100419 (2021).

[14] Schmitz, H. et al. Quantum walk of a trapped ion in phase space. *Phys. Rev. Lett.* **103**, 090504 (2009). 19792773 10.1103/PhysRevLett.103.090504

[15] Ramasesh, V. V., Flurin, E., Rudner, M., Siddiqi, I. & Yao, N. Y. Direct probe of topological invariants using Bloch oscillating quantum walks. *Phys. Rev. Lett.* **118**, 130501 (2017). 28409954 10.1103/PhysRevLett.118.130501

[16] Su, Q.-P. et al. Experimental demonstration of quantum walks with initial superposition states. *npj Quantum Inf.* **5**, 40 (2019).

[17] Gräfe, M. & Szameit, A. Integrated photonic quantum walks. *J. Phys. B* **53**, 073001 (2020).

[18] Esposito, C. et al. Quantum walks of two correlated photons in a 2D synthetic lattice. *npj Quantum Inf.* **8**, 34 (2022).

[19] Qiang, X. et al. Implementing graph-theoretic quantum algorithms on a silicon photonic quantum walk processor. *Sci. Adv.* **7**, eabb8375 (2021). 33637521 10.1126/sciadv.abb8375 PMC7909884

[20] Yuan, L., Lin, Q., Xiao, M. & Fan, S. Synthetic dimension in photonics. *Optica* **5**, 1396–1405 (2018).

[21] Cardano, F. et al. Quantum walks and wavepacket dynamics on a lattice with twisted photons. *Sci. Adv.* **1**, e1500087 (2015). 26601157 10.1126/sciadv.1500087 PMC4643825

[22] Imany, P., Lingaraju, N. B., Alshaykh, M. S., Leaird, D. E. & Weiner, A. M. Probing quantum walks through coherent control of high-dimensionally entangled photons. *Sci. Adv.* **6**, eaba8066 (2020). 32832628 10.1126/sciadv.aba8066 PMC7439509

[23] Schreiber, A. et al. Photons walking the line: a quantum walk with adjustable coin operations. *Phys. Rev. Lett.* **104**, 050502 (2010). 20366754 10.1103/PhysRevLett.104.050502

[24] Brendel, J., Gisin, N., Tittel, W. & Zbinden, H. Pulsed energy–time entangled twin-photon source for quantum communication. *Phys. Rev. Lett.* **82**, 2594 (1999).

[25] Reimer, C. et al. Generation of multiphoton entangled quantum states by means of integrated frequency combs. *Science* **351**, 1176–1180 (2016). 26965623 10.1126/science.aad8532

[26] Reimer, C. et al. High-dimensional one-way quantum processing implemented on d-level cluster states. *Nat. Phys.* **15**, 148–153 (2019).

[27] Ono, T., Tsujimoto, Y., Wakui, K. & Fujiwara, M. Quantum interference of pulsed time-bin entanglement generated from silicon ring resonator. *Sci Rep.* **14**, 1051 (2024). 38200214 10.1038/s41598-024-51311-4 PMC10781760

[28] Regensburger, A. et al. Parity–time synthetic photonic lattices. *Nature* **488**, 167–171 (2012). 22874962 10.1038/nature11298

[29] Bartlett, B., Dutt, A. & Fan, S. Deterministic photonic quantum computation in a synthetic time dimension. *Optica* **8**, 1515–1523 (2021).

[30] Wimmer, M., Miri, M.-A., Christodoulides, D. & Peschel, U. Observation of Bloch oscillations in complex PT-symmetric photonic lattices. *Sci Rep.* **5**, 17760 (2015). 26639941 10.1038/srep17760 PMC4671013

[31] Wimmer, M., Dhinwa, M., Carusotto, I., Peschel, U. & Price, H. M. Superfluidity of light and its breakdown in optical mesh lattices. *Phys. Rev. Lett.* **127**, 163901 (2021). 34723580 10.1103/PhysRevLett.127.163901

[32] Ozawa, T. & Price, H. M. Topological quantum matter in synthetic dimensions. *Nat. Rev. Phys.* **1**, 349–357 (2019).

[33] Motes, K. R., Gilchrist, A., Dowling, J. P. & Rohde, P. P. Scalable boson sampling with time-bin encoding using a loop-based architecture. *Phys. Rev. Lett.* **113**, 120501 (2014). 25279613 10.1103/PhysRevLett.113.120501

[34] Rohde, P. P. Simple scheme for universal linear-optics quantum computing with constant experimental complexity using fiber loops. *Phys. Rev. A* **91**, 012306 (2015).

[35] Rohde, P. P., Schreiber, A., Štefaňák, M., Jex, I. & Silberhorn, C. Multi-walker discrete time quantum walks on arbitrary graphs, their properties and their photonic implementation. *New J. Phys.* **13**, 013001 (2011).

[36] Jayakody, M. N., Pradhan, P., Ben Porath, D. & Cohen, E. Discrete-time quantum walk on multilayer networks. *Entropy* **25**, 1610 (2023). 38136490 10.3390/e25121610 PMC10743082

[37] Martin, A. et al. Cross time-bin photonic entanglement for quantum key distribution. *Phys. Rev. A* **87**, 020301 (2013).

[38] Kim, J.-H., Chae, J.-W., Jeong, Y.-C. & Kim, Y.-H. Quantum communication with time-bin entanglement over a wavelength-multiplexed fiber network. *APL Photonics* **7**, 016106 (2022).

[39] Kues, M. et al. On-chip generation of high-dimensional entangled quantum states and their coherent control. *Nature* **546**, 622–626 (2017). 28658228 10.1038/nature22986

[40] Franson, J. D. Bell inequality for position and time. *Phys. Rev. Lett.* **62**, 2205–2208 (1989). 10039885 10.1103/PhysRevLett.62.2205

[41] Collins, D., Gisin, N., Linden, N., Massar, S. & Popescu, S. Bell inequalities for arbitrarily high-dimensional systems. *Phys. Rev. Lett.* **88**, 040404 (2002). 11801097 10.1103/PhysRevLett.88.040404

[42] Guo, X., Mei, Y. & Du, S. Testing the Bell inequality on frequency-bin entangled photon pairs using time-resolved detection. *Optica* **4**, 388–392 (2017).

[43] Chang, K.-C. et al. 648 Hilbert-space dimensionality in a biphoton frequency comb: entanglement of formation and Schmidt mode decomposition. *npj Quantum Inf.* **7**, 48 (2021).

[44] Bacco, D. et al. Boosting the secret key rate in a shared quantum and classical fibre communication system. *Commun. Phys.* **2**, 140 (2019).

[45] Nitsche, T. et al. Quantum walks with dynamical control: graph engineering, initial state preparation and state transfer. *New J. Phys.* **18**, 063017 (2016).

[46] Deng, Y.-H. et al. Gaussian boson sampling with pseudo-photon-number-resolving detectors and quantum computational advantage. *Phys. Rev. Lett.* **131**, 150601 (2023). 37897783 10.1103/PhysRevLett.131.150601

[47] Kurzyński, P. & Wójcik, A. Quantum walk as a generalized measuring device. *Phys. Rev. Lett.* **110**, 200404 (2013). 25167387 10.1103/PhysRevLett.110.200404

[48] Wang, X. et al. Generalized quantum measurements on a higher-dimensional system via quantum walks. *Phys. Rev. Lett.* **131**, 150803 (2023). 37897782 10.1103/PhysRevLett.131.150803

[49] Annabestani, M., Hassani, M., Tamascelli, D. & Paris, M. G. A. Multiparameter quantum metrology with discrete-time quantum walks. *Phys. Rev. A* **105**, 062411 (2022).

