# Supplementary material for: Quantum state processing through controllable synthetic temporal photonic lattices
Source: Nat Photonics. 2024 Oct 14;19(1):95–100. doi: 10.1038/s41566-024-01546-4 (PMC11706774; doi:10.1038/s41566-024-01546-4)
Supplement: Supplementary file 1 — Supplementary Section 1. DTQW. Supplementary Section 2. QW as a measurement device. Supplementary Section 3. Uncontrolled and controlled DTQW evolutions to perform two- and four-level biphoton quantum interference measurements. Supplementary Section 4. Two-photon quantum interference for arbitrary discrete dimensions using TPLs. Supplementary Section 5. An exemplary application of TPLs: single phase estimation via synthetic TPLs. [file 41566_2024_1546_MOESM1_ESM.pdf]

nature photonics

Supplementary information

https://doi.org/10.1038/s41566-024-01546-4

# Quantum state processing through controllable synthetic temporal photonic lattices

In the format provided by the authors and unedited

**Supplementary information**

**Table of contents**

**S1. Discrete-time quantum walk.**

**S2. Quantum walk as a measurement device.**

**S3. Uncontrolled and controlled DTQW evolutions to perform two- and four-level biphoton quantum interference measurements.**

**S4. Two-photon quantum interference for arbitrary discrete dimensions using TPLs.**

**S5. An exemplary application of TPLs: single phase estimation via synthetic temporal photonic lattices.**

**S1. Discrete-time quantum walk.**

A discrete-time quantum walk (DTQW)[1–3] describes the dynamics of photons on a one-dimensional temporal photonic lattice (TPL)[4]. Many photonic platforms have been developed to implement DTQW using different optical degrees of freedom to encode the coin operation and the position of the quantum walker[5–7]. Each mode of the DTQW, $|\kappa\rangle := |n\rangle_{\boldsymbol{p}} \, |\sigma\rangle_{\boldsymbol{c}}$ , comprises two degrees of freedom, which are the synthetic position $|n\rangle_p$ and the coin state $|\sigma\rangle_{\boldsymbol{c}}$ with basis states $\{|\mathrm{S}\rangle_{\boldsymbol{c}}, |\mathrm{L}\rangle_{\boldsymbol{c}}\}$, where $|\mathrm{S}\rangle$ and $|\mathrm{L}\rangle$ represent the short and the long paths in the loops, respectively. The synthetic position ($n$) is encoded into the different arrival times of photons, which are dictated by the different lengths of the long and short loops. As we will see in the following sections, the temporal separation of the time bins (synthetic position) of the initial state of the quantum walkers must match the different lengths of the loops for quantum interference to happen. When representing pulses propagating in both loops in the same lattice, time positions alternate between the loops. The state of the quantum walker can be written as a superposition of the quantum modes $|\Psi(m)\rangle = \sum_\kappa \alpha_\kappa\,(m)|\kappa\rangle$, where $\alpha_\kappa(m)$ is the probability amplitude of finding the quantum walker in the mode $|\kappa\rangle$ at step $m$. The single-step quantum walk evolution $\widehat{U}_m = \hat{S} \cdot \hat{C}(n,m)$ is given by the product of the shift and the coin operators, namely $\hat{S}$ and $\hat{C}(n,m)$, respectively. Here, the shift operator moves the quantum walker according to its coin, as described in Eq. (1) [8]

$$\hat{S} = \sum_m |n+1\rangle_{\boldsymbol{p}} \, \langle n|_{\boldsymbol{p}} \otimes |\mathrm{L}\rangle_{\boldsymbol{c}}\langle \mathrm{L}|_{\boldsymbol{c}} + |n-1\rangle_{\boldsymbol{p}}\langle n|_{\boldsymbol{p}} \otimes |\mathrm{S}\rangle_{\boldsymbol{c}}\langle \mathrm{S}|_{\boldsymbol{c}}, \tag{1}$$

which mimics the different delays that photons experience in different loops. The coin operator has the form $\hat{C}(n,m) = \sum_n |n\rangle\,\langle n| \otimes \hat{C}(n,m)$, where[8]

$$\hat{C}(n,m) = \begin{pmatrix} \cos\theta_{n,m} & \mathrm{i}\sin\theta_{n,m} \\ \mathrm{i}\sin\theta_{n,m} & \cos\theta_{n,m} \end{pmatrix}, \tag{2}$$

as written in the coin bases $|\sigma\rangle_{\boldsymbol{c}} = \{|\mathrm{S}\rangle_{\boldsymbol{c}}, |\mathrm{L}\rangle_{\boldsymbol{c}}\}$. Here, the angle $\theta_{n,m}$ is responsible for the transmission and reflection coefficients. In our scheme, the coin operator is experimentally realized by an ultra-fast optical dynamic coupler (referred in the main text as the central coupler), which simulates various operations within different time bins and roundtrips for various transmission/reflection ratios. Specifically, the central coupler can be configured to perform full transmission $\hat{T}(n,m)$ (with $\theta_{n,m} = \pi/2$), which sends the walker from the short (long) to the long (short) loops, the 50:50 splitting (the Fourier coin) $\hat{F}(n,m)$ (with $\theta_{n,m} =$

$\pi/4$), which puts the walker in the superposition of the two loops, and the full reflection $\hat{R}(n,m)$ (with $\theta_{n,m} = 0$) , which keeps the walker in the same loop. It is worth recalling that the single-step unitary evolution of two quantum walkers is given[9] by $\hat{\mathcal{U}}_m = \hat{\mathcal{U}}_m^s \otimes \hat{\mathcal{U}}_m^i$, where the first operator acts on the signal ($s$) and the second on the idler ($i$) photon. The evolution of the quantum walker after the $m$-th step is given by $\hat{\mathcal{U}} = \Pi_{j=1}^{m} \hat{\mathcal{U}}_j$, which provides $m$ repetitive actions for the independent single-step unitary evolution of two quantum walkers.

**S2. Quantum walk as a measurement device.**

It has been shown that a DTQW can be used to implement a generalized measurement[10] in terms of a positive operator value measure (POVM)[11], $\sum_i \hat{E}_i = \hat{\mathbb{I}}$, with $\hat{\mathbb{I}}$ denoting the identity operator and $\hat{E}_i$ representing POVMs. A general quantum walk evolution $\hat{\mathcal{U}} = \sum_i |i\rangle \langle\psi_{inp}|$ maps the input (initial) state $|\psi_{inp}\rangle$ into the final state $|i\rangle$. As such, projective measurements $\hat{M}_i = |i\rangle\langle i|$ on the final state correspond to POVMs on the initial state. The probability of measuring the $i^{\text{th}}$ POVM element on a quantum state density matrix $\hat{\rho}$ is $p_i = \mathrm{Tr}(\hat{E}_i \hat{\rho})$, with Tr labeling the trace. The intended POVM that leads to quantum interference measurement is $\hat{E}_{\mathrm{p}} = |\psi_{\mathrm{p}}\rangle\langle\psi_{\mathrm{p}}|$, with[12]

$$|\psi_{\mathrm{p}}\rangle = \frac{1}{d}\left(\sum_{t=0}^{d-1} e^{\mathrm{i}t\theta} |t\rangle_s\right)\left(\sum_{t=0}^{d-1} e^{\mathrm{i}t\theta} |t\rangle_i\right), \tag{3}$$

where $d$ is the number of time bins per single photon (that is, the time-bin dimensionality per photon). Thus, the expected quantum interference is

$$\mathcal{P}_d = |\langle\psi_{\mathrm{d}}|\psi_{\mathrm{p}}\rangle|^2 = \frac{1}{d^3}\left(\frac{\sin^2(d\theta)}{\sin^2(\theta)}\right). \tag{4}$$

To retrieve the quantum interference pattern, we let the system evolve according to a controlled unitary evolution for $m$ roundtrips as $\psi_{\mathrm{d}}(m) = \Pi_{j=1}^{m} \hat{\mathcal{U}}_j \psi_{\mathrm{d}}(0)$ . Then, the projection measurement $\hat{M} = |-1\mathrm{S}\rangle_s|-1\mathrm{S}\rangle_i\langle-1\mathrm{S}|_s\langle-1\mathrm{S}|_i$ is applied, corresponding to the coincidence between the signal and idler photons at the synthetic position (i.e., the time mode) $n = -1$ in the short loop corresponding to the mode $|-1\rangle_{\boldsymbol{p}}|\mathrm{S}\rangle_{\boldsymbol{c}}$. Therefore, the expected quantum interference can be rewritten as

$$\mathcal{P}_d = \langle\psi_{\mathrm{d}}(m)|\hat{M}|\psi_{\mathrm{d}}(m)\rangle/p_0^d \tag{5}$$

where $p_0^d \in [0, 1]$ is a constant factor, whose range is the same as the detection efficiency. Therefore, $p_0$ allows quantum walk evolutions with different detection efficiencies, which leads, in turn, to difference quantum interference patterns.

## S3. Uncontrolled and controlled DTQW evolutions to perform two- and four-level biphoton quantum interference measurements.

We provide a theoretical framework for different evolution operators of the two-photon states to realize quantum interference measurements for both 2-level and 4-level input states, as shown in the main text. We employed two main DTQW strategies: an uncontrolled scheme, in which the coupler is fixed at 50:50 splitting ratio, and a controlled scheme, in which the coupler was dynamically changed between full transmission, full reflection, and 50:50 splitting ratio. For the four-level case, the latter scheme, in turn, can be divided into two categories: an inter-roundtrip scheme (control scheme 1) and an intra-roundtrip scheme (control scheme 2).

For the two-level case, we inject the qubit state $|\psi_2(0)\rangle = \frac{1}{\sqrt{2}}(|-1\mathrm{S}\rangle_s|-1\mathrm{S}\rangle_i + e^{2i\theta}|1\mathrm{S}\rangle_s|1\mathrm{S}\rangle_i)$ into the system. Afterward, we let it evolve into two roundtrips with the uncontrolled DTQW scheme, in which the 50:50 coupling ratio is described by the Fourier coin $\hat{F}_{s(i)}$, see Fig. 3 (A) of the main text. The total unitary operator is thus given by

$$\hat{\mathcal{U}}_F = \hat{\mathcal{S}}_s(\hat{\mathbb{I}}_{\boldsymbol{p}} \otimes \hat{F}_s) \otimes \hat{\mathcal{S}}_i(\hat{\mathbb{I}}_{\boldsymbol{p}} \otimes \hat{F}_i), \tag{6}$$

where $\hat{\mathbb{I}}_p$ is the identity over the position space. Thus, after these two steps, the evolved state is $|\psi_2(m=2)\rangle = \hat{\mathcal{U}}_F\hat{\mathcal{U}}_F|\psi_2(0)\rangle$. The expected interference in Eq. 4 is obtained using the measurement outcome as $\mathcal{P}_2 = \langle\psi_2'(m=2)|\hat{M}|\psi_2'(m=2)\rangle/p_0^{d=2}$, where $p_0^{d=2} = 1/4$ is the detection efficiency. However, the quantum system is inevitably affected by noise contribution from the measurement settings. Therefore, one can consider the white noise model[13] as $\hat{\rho}(m) = \epsilon_m|\psi(m)\rangle\langle\psi(m)| + \frac{(1-\epsilon_m)}{D}\hat{\mathbb{I}}$, where $\epsilon_m$ is the probability that the state $|\psi(m)\rangle$ is unaffected by noise, and $D$ is the number of modes that quantum walkers (here, the two entangled photons) occupy at step $m$ with nonzero probability amplitudes. The quantum interference reads[12]

$$\mathcal{P}_2(\epsilon_2) = \frac{1}{4}(1 + \epsilon_2\cos2\theta), \tag{7}$$

which is obtained by performing the projective measurement as $\mathcal{P}_2 = \mathrm{Tr}\left(\hat{M}\hat{\rho}(m=2)\right)$. It is worth recalling that Fig. 3 (C) of the main text shows the experimental quantum interference.

In the controlled DTQW scheme (Fig. 3 (D) of the main text), we let the input state $|\psi_2(0)\rangle$ evolve with two independent unitary operators. The operation for the first step is

$$\hat{\mathcal{U}}_{TR} = \hat{\mathcal{S}}_{\mathrm{s}}\cdot\left(\sum_{n=0}\left(|-n\rangle_{\boldsymbol{p}}\langle -n|_{\boldsymbol{p}} \otimes \hat{T}_s + |n\rangle_{\boldsymbol{p}}\langle n|_{\boldsymbol{p}} \otimes \hat{R}_s\right)\right) \otimes \hat{\mathcal{S}}_{\mathrm{i}}\cdot\left(\sum_{n=0}\left(|-n\rangle_{\boldsymbol{p}}\langle -n|_{\boldsymbol{p}} \otimes \hat{T}_i + |n\rangle_{\boldsymbol{p}}\langle n|_{\boldsymbol{p}} \otimes \hat{R}_i\right)\right), \quad (8)$$

which sends the photons at the earlier time bin (synthetic position $n$ = -1) into the long loop with the transmission coin $\hat{T}_{s(i)}$ and keeps those at the latter time bin (synthetic position $n$ = +1) into the short loop using $\hat{R}_{s(i)}$. In the next step, we let the photons interfere (at the central coupler, corresponding to the synthetic position $n$ = 0) with 50:50 ratio coupling given in Eq. 6. Thus, after these two steps, the evolved state is $|\psi_2(m=2)\rangle = \hat{\mathcal{U}}_F\hat{\mathcal{U}}_{TR}|\psi_2(0)\rangle$. Finally, the quantum interference described in Eq. 5 with $d$ = 2 is obtained by performing the projective measurement $\hat{M}$. As discussed in the main text, the controlled DTQW scheme enables increasing the number of coincidence counts and therefore maximizing the detection efficiency to $p_0^{d=2} = 1$. The slightly different visibilities measured in our experiment, i.e., $V_{d=2}$ = 97.82% and $V'_{d=2}$ = 96.83% for the controlled and uncontrolled cases, respectively, can be justified as different loss amounts affecting the two evolution schemes due to the executions of different operations.

The scalability of our platform allows us to perform DTQWs with a higher number of (entangled) time bins as input states. Specifically, we could inject a four-level entangled state of the form $|\psi_4\rangle = \frac{1}{2}(|-3\mathrm{S}\rangle_s|-3\mathrm{S}\rangle_i + e^{2\mathrm{i}\theta}|-1\mathrm{S}\rangle_s|-1\mathrm{S}\rangle_i + e^{4\mathrm{i}\theta}|1\mathrm{S}\rangle_s|1\mathrm{S}\rangle_i + e^{6\mathrm{i}\theta}|3\mathrm{S}\rangle_s|3\mathrm{S}\rangle_i)$ into the loop system. Two different evolution strategies are designed to perform the quantum interference measurement described by Eq. 5.

In control scheme 1, shown in Fig. 4 (A) of the main text, the central coupler is set to 50:50 ( $\hat{F}$) in the first two roundtrips, to full reflection (R) in the third roundtrip, and finally to 50:50 ($\hat{F}$) in the fourth roundtrip. The unitary evolution is given by $\mathcal{U}_{\mathrm{d}=4} = \hat{\mathcal{U}}_F\hat{\mathcal{U}}_R\hat{\mathcal{U}}_F\hat{\mathcal{U}}_F$, composed of two 50:50 splitting operations in the first two roundtrips, a pure reflection as

$$\hat{\mathcal{U}}_R = \hat{\mathcal{S}}_s(\mathbb{I}_{\boldsymbol{p}} \otimes \hat{R}_s) \otimes \hat{\mathcal{S}}_i(\mathbb{I}_{\boldsymbol{p}} \otimes \hat{R}_i) \quad (9)$$

in the third roundtrip, and another 50:50 splitting operation at the last roundtrip. This procedure enabled us to extract the photons from the short loop and post-select the central bin out of seven bins. Quantum interference, given in Eq. 2 with $d$ = 4, is finally obtained by performing projections of the form $\mathcal{P}_4 = \langle\psi_4(m=4)|\widehat{M}|\psi_4(m=4)\rangle/p_0^{d=4}$, where $|\psi_4(m=4)\rangle = \mathcal{U}_{\mathrm{d=4}}|\psi_4(0)\rangle$ is the evolved state and $p_0^{d=4} = 1/4$.

In control scheme 2, shown in Fig. 4 (C) of the main text, the central coupler is set to transmit the first two time bins (or synthetic position $n$=-3 and $n$=-1) into the long loop, and to reflect the other two time bins (or synthetic position $n$=1 and $n$=3) within the short loop. In the second roundtrip, the coupler is set to reflect all the four bins within the loop in which they were in the previous step. Finally, in the last two roundtrips, the coupler is set to 50:50, to make the bins interfere in a given coincidence window (see Fig. 4 (C) of the main text). This procedure enabled us to extract the photons from the short loop and post-select the central bin out of only three bins (instead of seven bins). The unitary operator of these mentioned roundtrips is given by $\mathcal{U}_{\mathrm{d=4}} = \widehat{\mathcal{U}}_F\widehat{\mathcal{U}}_F\widehat{\mathcal{U}}_R\widehat{\mathcal{U}}_{TR}$. Finally, the expected quantum interference pattern is given by the projective measurement $\mathcal{P}_4 = \langle\psi_4'(m=4)|\widehat{M}|\psi_4'(m=4)\rangle$, where $|\psi_4'(m=4)\rangle = \widehat{\mathcal{U}}_{d=4}|\psi_4(0)\rangle$ is the evolved state. The quantum interference pattern of a four-level input state is given as[12]

$$\mathcal{P}_4(\epsilon_4) = \frac{1}{2^6}\left(4 + 2\epsilon_4(3\cos 2\theta + 2\cos 4\theta + \cos 6\theta)\right), \tag{10}$$

which is obtained by assuming the white noise model introduced earlier. As shown in the main text, the imperfect behavior of the dynamical coupler increases the noise ratio. This affects the visibility, which declines from $V_{d=4}$ = 91.55% to $V'_{d=4}$ = 89.61%.

**S4. Two-photon quantum interference for arbitrary discrete dimensions using TPLs.**

Although the experimental realization of the two-photon quantum interference measurement is limited to 4 levels, the proposed measurement scheme can be generalized to more than four level with $d = 2^N$ $(N = 1, \ldots)$. We assume that the process starts with sending the input state through the short loop, then adjusting the dynamics via the central coupler to implement the projection of Eq. 3. The first rule is that we need $d$ roundtrips to verify two-photon quantum interference of the $d$-level input state. Specifically, $d$ independent unitary operators $\widehat{\mathcal{U}}_d = \Pi_{j=1}^{d}\widehat{\mathcal{U}}_j$ are constructed to make the initial state evolve as $|\psi_d(m=d)\rangle = \widehat{\mathcal{U}}_d|\psi_d(0)\rangle$. Then, the projection measurement $\widehat{M}$ is applied to achieve the quantum interference of Eq. 4. The

process begins by applying the unitary operator $\hat{\mathcal{U}}_{TR}$ (given in Eq. 6) followed by $(d/2\ -1)$ pure reflection operators (given in Eq. 9). In the time-bin mixing stage, we let the photons evolve with different sequences of the 50:50 splitting and reflection operators, presented below for up to 16 levels

$$d=2:\quad \mathcal{U}_{\mathrm{d}=2}=\hat{\mathcal{U}}_F\hat{\mathcal{U}}_{TR}$$

$$d=4:\quad \mathcal{U}_{\mathrm{d}=4}=\left(\hat{\mathcal{U}}_F\right)^2\hat{\mathcal{U}}_R\hat{\mathcal{U}}_{TR}$$

$$d=8:\quad \mathcal{U}_{\mathrm{d}=8}=\hat{\mathcal{U}}_F\hat{\mathcal{U}}_R\left(\hat{\mathcal{U}}_F\right)^2\left(\hat{\mathcal{U}}_R\right)^3\hat{\mathcal{U}}_{TR}$$

$$d=16:\quad \mathcal{U}_{\mathrm{d}=16}=\hat{\mathcal{U}}_F\left(\hat{\mathcal{U}}_R\right)^3\hat{\mathcal{U}}_F\hat{\mathcal{U}}_R\left(\hat{\mathcal{U}}_F\right)^2\left(\hat{\mathcal{U}}_R\right)^7\hat{\mathcal{U}}_{TR}. \tag{11}$$

Finally, we take photons out from the short loop and performed coincidence measurements at the synthetic position $n=-1$ for different values of $\theta$, thus obtaining the expected quantum interference in Eq. 4.

**S5. An exemplary application of TPLs: single phase estimation via synthetic temporal photonic lattices.**

In quantum metrology, such as phase estimation problems, entangled states provide enhanced phase sensitivity – known as the Heisenberg limit ($\delta\varphi \geq 1/\sqrt{\mu N^2}$) – which surpasses the classical shot noise limit[14] ($\delta\varphi \geq 1/\sqrt{\mu N}$). Here, $\mu$ represents the number of experimental repetitions and $N$ is the number of particles, like photons, used in the experiments. In addition to using entangled states, the highest estimation accuracy can be obtained through optimal positive operator-valued measures (POVMs), a complete set of projective measurements that are sufficient to determine the probabilities of the different measurement outcomes[11]. POVMs applied to entangled states thus lead to saturate the so-called Quantum Cramer-Rao bound, given by $\delta\varphi \geq 1/\sqrt{\mu F}$, where $F$ is the quantum Fisher information (QFI)[15].

The controllability of the TPL motivates us to design an optimal measurement strategy (i.e., POVMs) to estimate an unknown phase encoded in a biphoton time-bin entangled initial state of the form $|\psi\rangle = \frac{1}{\sqrt{2}}\left(|0,0\rangle_{s,i} + e^{2i\varphi}|1,1\rangle_{s,i}\right)$. Here, the parameter to be estimated is the unknown phase difference $\varphi$ between the early (0) and late (1) time bins of the two photons (signal and idler). In ideal conditions, the state $|\psi\rangle$ can potentially lead to a QFI equal to $F=4$. To achieve such sensitivity ($\delta\varphi \geq 1/\ 2\sqrt{\mu}$), we can design the DTQW evolution in such a

way to perform the desired optimal POVMs. To this end, similarly to the strategy described in the main text for quantum interference measurements, we inject the initial state $|\psi\rangle$ to the TPL. Afterwards, we perform the controlled DTQW scheme, illustrated in Fig. 1 (B), and let it evolve over two roundtrips through the two unitary operators given in Eq. 11. Finally, we can apply the projective measurements in both positions $x = -1$ and $x = 1$, to thus obtain four DTQW probability outputs: $P_{-1,-1}(\varphi) = P_{1,1}(\varphi) = \frac{1+\cos 2\varphi}{4}$ and $P_{1,-1}(\varphi) = P_{1,-1}(\varphi) = \frac{1-\cos 2\varphi}{4}$. $P_{-1,-1}$ and $P_{1,1}$ denote the probability that both photons exit from the long (short) loop, while $P_{1,-1}$ and $P_{-1,1}$ denote the probability that one photon exits from the long loop and the other from the short loop (and vice versa). Afterwards, measuring the QFI in terms of probabilities as[15] $F(\varphi) = \sum_i \frac{[\partial_\varphi P_i(\varphi)]^2}{P_i(\varphi)}$, we can obtain $F = 4$. This clearly shows how TPL can be utilized to implement an optimal measurement strategy.

## References


1. Lovett, N. B., Cooper, S., Everitt, M., Trevers, M. & Kendon, V. Universal quantum computation using the discrete-time quantum walk. *Phys. Rev. A* **81**, 042330 (2010).
2. Jayakody, M. N., Pradhan, P., Ben Porath, D. & Cohen, E. Discrete-time quantum walk on multilayer networks. *Entropy* **25**, 1610 (2023).
3. Chawla, P., Singh, S., Agarwal, A., Srinivasan, S. & Chandrashekar, C. M. Multi-qubit quantum computing using discrete-time quantum walks on closed graphs. *Sci. Rep.* **13**, 12078 (2023).
4. Regensburger, A. *et al.* Parity-time synthetic photonic lattices. *Nature* **488**, 167–171 (2012).
5. Schreiber, A. *et al.* Photons walking the line: a quantum walk with adjustable coin operations. *Phys. Rev. Lett.* **104**, 50502–50502 (2010).
6. Broome, M. A. *et al.* Discrete single-photon quantum walks with tunable decoherence. *Phys. Rev. Lett.* **104**, 153602–153602 (2010).

7. Cardano, F. *et al.* Quantum walks and wavepacket dynamics on a lattice with twisted photons. *Sci. Adv.* **1**, e1500087–e1500087 (2015).

8. Venegas-Andraca, S. E. Quantum walks: a comprehensive review. *Quantum Inf. Process.* **11**, 1015–1106 (2012).

9. Omar, Y., Paunković, N., Sheridan, L. & Bose, S. Quantum walk on a line with two entangled particles. *Phys. Rev. A* **74**, 042304 (2006).

10. Kurzyński, P. & Wójcik, A. Quantum walk as a generalized measuring device. *Phys. Rev. Lett.* **110**, 200404–200404 (2013).

11. Nielsen, M. A. & Chuang, I. L. *Quantum Computation and Quantum Information*. (Cambridge University Press, 2010).

12. Kues, M. *et al.* On-chip generation of high-dimensional entangled quantum states and their coherent control. *Nature* **546**, 622–626 (2017).

13. Collins, D., Gisin, N., Linden, N., Massar, S. & Popescu, S. Bell inequalities for arbitrarily high-dimensional systems. *Phys. Rev. Lett.* **88**, 40404–40404 (2002).

14. Giovannetti, V., Lloyd, S. & MacCone, L. Advances in quantum metrology. *Nat. Photonics* **5**, 222–229 (2011).

15. Paris, M. G. Quantum estimation for quantum technology. *Int. J. Quantum Inf.* **7**, 125–137 (2009).
